# *Ex Vivo* Regional Gene Therapy Compared to Recombinant BMP-2 for the Treatment of Critical-Size Bone Defects: An In Vivo Single-Cell RNA-Sequencing Study

**DOI:** 10.3390/bioengineering12010029

**Published:** 2025-01-01

**Authors:** Arijita Sarkar, Matthew C. Gallo, Jennifer A. Bell, Cory K. Mayfield, Jacob R. Ball, Mina Ayad, Elizabeth Lechtholz-Zey, Stephanie W. Chang, Osamu Sugiyama, Denis Evseenko, Jay R. Lieberman

**Affiliations:** 1 Department of Orthopaedic Surgery, Keck School of Medicine of the University of Southern California, Los Angeles, CA 90033, USA; matthew.gallo@med.usc.edu (M.C.G.); jennifer.bell@med.usc.edu (J.A.B.); cory.mayfield@med.usc.edu (C.K.M.); jacob.ball@med.usc.edu (J.R.B.); mina.ayad2@med.usc.edu (M.A.); elizabeth.lechtholz-zey@med.usc.edu (E.L.-Z.); changsw@usc.edu (S.W.C.); osugiyam@med.usc.edu (O.S.); evseenko@usc.edu (D.E.); jay.lieberman@med.usc.edu (J.R.L.); 2 Alfred E. Mann Department of Biomedical Engineering, Viterbi School of Engineering, University of Southern California, Los Angeles, CA 90089, USA

**Keywords:** single-cell RNA sequencing, gene therapy, BMP-2, mesenchymal stem cells, endochondral ossification, inflammation, bone defect, bone regeneration

## Abstract

*Ex vivo* regional gene therapy is a promising tissue-engineering strategy for bone regeneration: osteogenic mesenchymal stem cells (MSCs) can be genetically modified to express an osteoinductive stimulus (e.g., bone morphogenetic protein-2), seeded onto an osteoconductive scaffold, and then implanted into a bone defect to exert a therapeutic effect. Compared to recombinant human BMP-2 (rhBMP-2), which is approved for clinical use, regional gene therapy may have unique benefits related to the addition of MSCs and the sustained release of BMP-2. However, the cellular and transcriptional mechanisms regulating the response to these two strategies for BMP-2 mediated bone regeneration are largely unknown. Here, for the first time, we performed single-cell RNA sequencing (10x Genomics) of hematoma tissue in six rats with critical-sized femoral defects that were treated with either regional gene therapy or rhBMP-2. Our unbiased bioinformatic analysis of 2393 filtered cells in each group revealed treatment-specific differences in their cellular composition, transcriptional profiles, and cellular communication patterns. Gene therapy treatment induced a more robust chondrogenic response, as well as a decrease in the proportion of fibroblasts and the expression of profibrotic pathways. Additionally, gene therapy was associated with an anti-inflammatory microenvironment; macrophages expressing canonical anti-inflammatory markers were more common in the gene therapy group. In contrast, pro-inflammatory markers were more highly expressed in the rhBMP-2 group. Collectively, the results of our study may offer insights into the unique pathways through which *ex vivo* regional gene therapy can augment bone regeneration compared to rhBMP-2. Furthermore, an improved understanding of the cellular pathways involved in segmental bone defect healing may allow for the further optimization of regional gene therapy or other bone repair strategies.

## 1. Introduction

Large bone defects may occur in the setting of high-energy trauma, revision total joint replacement, or tumor resection [1]. While bone has a significant regenerative capacity, defects beyond a certain size may fail to heal spontaneously despite surgical stabilization. These defects require additional surgical intervention. Current treatment strategies focus on restoring local biology by providing one or more components including osteogenic cells, osteoinductive growth factors, or an osteoconductive scaffold [1,2]. An autologous bone graft is considered the gold standard treatment as it provides all three [3]. However, there are limitations including the volume of available graft, donor site morbidity, and inconsistent clinical results in larger defects [3]. Thus, identifying alternative therapeutic strategies for bone loss scenarios is of great clinical importance.

Bone morphogenetic protein 2 (BMP-2) is a member of the transforming growth factor beta (*TGFB*) superfamily and plays a critical role in osteogenesis and fracture healing [4,5,6]. The recombinant form of the protein, rhBMP-2, is a potent osteoinductive agent that is FDA-approved for use in open tibia fractures, anterior lumbar spine fusion, and maxillofacial augmentation [7]. While early results were promising, widespread clinical adoption was associated with significant adverse effects including soft tissue swelling, bone resorption, and abnormal bone formation [8]. It was hypothesized that these off-target effects were related to the protein’s rapid diffusion away from the collagen carrier, limiting its duration of effect, as well as the supraphysiologic doses of rhBMP-2 required to induce adequate bone formation [8,9,10]. Therefore, there is great interest in improving the delivery mechanisms for BMP-2 to enhance the quality of bone repair [11].

*Ex vivo* regional gene therapy is a promising tissue-engineering strategy for bone regeneration that addresses the limitations with rhBMP-2 delivery. In *ex vivo* gene therapy approaches for bone repair, osteogenic cells, typically mesenchymal stem cell (MSCs), are first genetically modified in tissue culture to express an osteoinductive protein. The cells are subsequently harvested, loaded onto an osteoconductive scaffold, and then implanted at the site of bone loss to exert a therapeutic effect [12]. Prior gene therapy studies have shown BMP-2 expression *in vivo* for days to weeks depending on the type of vector chosen. In contrast, there is a “burst” delivery associated with rhBMP-2 loaded on a collagen sponge, which is how rhBMP-2 is used in clinical formulations [10,13]. In addition to sustained BMP-2 expression, *ex vivo* gene therapy has additional theoretical advantages including (1) the increased potency of transgenic BMP-2 compared to rhBMP-2 due to post-translational modification, (2) MSCs serving as a source of multipotent progenitors, and (3) MSCs possessing immunomodulatory properties that may favorably alter the bone healing microenvironment [14,15]. Over the past two decades, the efficacy of regional gene therapy for bone regeneration has become well-established in preclinical models. Our group and others have shown that MSCs modified to express BMP-2 induce consistent healing in rat critical-sized femoral defects. Furthermore, the newly formed bone is radiographically, histologically, and biomechanically comparable or superior to the bone formation seen with rhBMP-2 treatment [16]. Despite these promising preclinical results, the underlying cellular and molecular mechanisms of bone healing with regional gene therapy, as well as rhBMP-2, remain to be determined.

Single-cell RNA sequencing (scRNA-seq) has emerged as a powerful technology that enables a detailed examination of cellular and molecular diversity. In recent years, it has been applied to musculoskeletal biology, providing novel insights on cellular heterogeneity and cellular events that govern skeletal development, homeostasis, and disease states including osteoporosis, osteoarthritis, and fracture healing [17,18]. This technology has the potential to identify the molecular mechanisms driving orthopedic disease, which is a critical first step to developing effective interventions. To our knowledge, scRNA-seq technology has not previously been applied in the context of therapeutic interventions for bone loss, which may enable the identification of important pathways that provide maximal osteogenic effects while minimizing potential side effects.

In this study, we analyzed hematoma tissue from rats with critical-sized femoral defects treated with two strategies for BMP-2 mediated bone regeneration: *ex vivo* regional gene therapy utilizing adipose-derived MSCs transduced with a lentiviral vector containing the cDNA for BMP-2 and the regional administration of rhBMP-2. Using scRNA-seq, we identified treatment-specific cell populations, transcriptome characteristics, and cell communication patterns that suggest unique pathways through which a novel regional gene therapy approach may augment *in vivo* bone regeneration compared to rhBMP-2.

## 2. Materials and Methods

This study protocol was approved by the Institutional Animal Care and Use Committee (IACUC) and conducted in accordance with its guidelines, as well as the regulations set by the NIH for the care of research animals.

### 2.1. Adipose-Derived Stem Cell Isolation and Culture

Adipose tissue was collected from the peri-testicular fat of 8-week-old male Lewis rats (Charles River Laboratories, Wilmington, MA, USA) and the stromal vascular fraction (SVF) was obtained and processed using a previously published protocol [16,19]. The SVF including human adipose-derived mesenchymal stem cells (ASCs) was then resuspended in Iscove’s Modified Dulbecco’s Medium (IMDM; Corning Inc., Corning, NY, USA) which contained 15% fetal bovine serum (Gibco, Amarillo, TX, USA) and an antibiotic mix containing 100 unit/mL of penicillin, 100 μg/mL streptomycin, and 250 ng/mL Amphotericin B (Lonza, Basel, CH). The suspension was plated on a 100 mm dish for culture at a density of 2–3 × 10^6^ cells/plate. Culture media was changed every 3–4 days. Adherent ASCs were split weekly and plated at a density of 7–8 × 10^5^ cells/plate. Passage 3 cells were used for *in vivo* experiments.

### 2.2. Viral Vector Generation and Transduction

ASCs were transduced using a two-step transcriptional amplification (TSTA) lentiviral system overexpressing BMP-2 (LV-BMP-2), as per previously published protocols [20,21,22]. Briefly, the TSTA system contains two lentiviral vectors: GAL4-VP16 transactivator vector (LV-RhMLV- GAL4-VP16) and a transgene expression vector encoding BMP-2 (LV-G5-BMP-2). LV vectors were generated by transfecting 293 T cells (American Type Culture Collection, Manassas, VA, USA) using established techniques [23]. To quantify the titer of vectors, HT-29 cells (ATCC, Manassas, VA, USA) were transduced with diluted vector prep and genomic DNA was extracted at 3 days after transduction. Human cell number and lentiviral vector copy number were quantified using QX200 droplet digital PCR system (Bio-rad, Herclus, CA, USA) and HIV-1 psi region and human Syndecan-2 (SDC-4) primers and probes. Vector copy number per cell was used to determine LV titer.

At passage 3, ASCs were plated at a density of 1 × 10^6^ cells/plate. The following day cells were co-transduced with two LV vectors (LV-RhMLV-GAL4-VP16 and LV-G5-BMP-2) at MOI of 3 each, then incubated overnight. The following morning the transduced cells were washed with Dulbecco’s PBS to remove extracellular virus and incubated overnight in fresh media prior to implantation.

### 2.3. Rat Femoral Defect Model and Implantation of Therapeutic Products

The preclinical model used for this study was a 6 mm rat critical-sized femoral bone defect, which is a well-established model for studying bone healing [24,25,26]. A critical-sized defect is defined as a defect that will not heal spontaneously despite surgical stabilization.

Briefly, anesthesia was induced using inhalation of 2–3% isoflurane in an induction chamber and was maintained with a nose cone. The left hindlimb was prepped in sterile fashion. In a prone position, a posterolateral approach to the femur was taken, splitting quadriceps muscle and stripping periosteum circumferentially. A polyethylene plate was then secured to the femur using four-threaded Kirschner wires (Zimmer Biomet, Warsaw, IN, USA) and two stainless steel cerclage wires. A 6 mm bone defect was then created in the mid-diaphysis using a side-cutting burr. Following defect creation, the medullary canals were copiously flushed with sterile 0.9% saline solution to remove remaining osteoprogenitor cells.

LV-BMP-2 transduced cells were harvested, mixed with 30 μL of DPBS, and loaded via onto a commercially available compression-resistant matrix (Medtronics Inc., Memphis, TN, USA) measuring 6 × 3 × 3 mm. The loaded scaffold was then placed into the defect site. For animals in the rhBMP-2 group, 10 μg of rhBMP-2 was mixed with 30 μL of DPBS prior to scaffold loading. Following implantation, 4-0 Vicryl suture was used to close fascia and skin. Subcutaneous buprenorphine SR injection was given post-operatively for pain relief.

### 2.4. Experimental Groups

Lewis rats were assigned to one of two study groups: standard dose (5 × 10^6^ cells) transduced with LV-BMP-2 (group I, n = 3) or a standard dose (10 μg) of rhBMP-2 (group II, n = 3). The standard dose of 5 × 10^6^ transduced cells and 10 μg of rhBMP-2 were chosen because prior experiments from our lab have shown similar healing with these two treatments in the described animal model [16,27,28].

### 2.5. Tissue Harvest

Animals were sacrificed at 14 days after cell or rhBMP-2 implantation. Deep anesthesia was provided with 4% isoflurane. The left hindlimb was prepped in sterile fashion. In a prone position, a posterolateral approach to the femur was taken using the prior incision and dissection plane to expose the femur. In both treatment groups, the defect site was obscured by an induced pseudomembrane, similar to what is observed in the Masquelet technique [29]. Between the Kirschner wires nearest to the defect (a 9 mm span), membrane overlying the plate, bone, and scaffold was carefully harvested. Next, using sharp dissection, the scaffold was excised from the defect site.

### 2.6. Cell Isolation

Cells were prepared according to a previously established protocol [16]. Harvested tissue was washed extensively with DPBS to remove debris and red blood cells. Scissors were used to mechanically cut the tissue into smaller pieces and enzymatic digestion was then performed with 0.2% collagenase type 1A at 37 °C for 90 min. Scaffold specimens were also treated with 0.25% trypsin for 8 min to liberate cells that may have remained adhered to scaffold granules. The digested tissue was then filtered with a 70 μm strainer and washed with IMDM media. RBC lysis was performed using a commercial buffer (Lonza, Basel, CH). Cells were then stained with anti-RBC antibody according to manufacturer’s protocol (BioLegend, San Diego, CA, USA), washed, and resuspended in PBS with 1% FBS containing DAPI. Fluorescence-activated cell sorting (FACS) analysis was performed to identify and sort live cells using a BD LSR II flow cytometer (BD Biosciences, Franklin Lakes, NJ, USA). The gating strategy for FACS sorting has been provided in Supplementary Figure S1.

### 2.7. Single-Cell RNA Sequencing

Single-cell samples were prepared using the Chromium Next GEM Single Cell 3′ Reagent Kits v3 and Chip Kits (10x Genomics, Pleasanton, CA, USA), following the manufacturer’s protocol [30]. In brief, FACS-sorted live cells were resuspended in 0.04% BSA-PBS solution. Approximately 700–1000 cells/μL were added to each chip well, aiming for a target cell recovery of 5000–9000 cells. Gel bead-in emulsions (GEMs) were generated using the Chromium controller (10x Genomics, Pleasanton, CA, USA). GEMs underwent reverse transcription, the droplets were broken, and single-stranded cDNA was isolated. The cDNA was cleaned using DynaBeads and amplified. After amplification, adapters were ligated to the cDNA, followed by further amplification and cleanup with SPRIselect. The quality of the purified libraries was checked using Agilent Tapestation D1000 (Agilent, Santa Clara, CA, USA) and their concentration was quantified by dsDNA high-sensitivity kit using Qubit fluorometer (Thermo Fisher Scientific, MA, USA). Equimolar concentration of the libraries was pooled and sequenced on Illumina NextSeq platform (Illumina, San Diego, CA, USA) at our institutional molecular genomics core laboratory.

### 2.8. Single-Cell RNA-Sequencing Data Analysis

Raw fastq files were checked for their quality and trimmed, and reads with an average base quality score per position > 30 was considered for further analysis using Cell Ranger (v7, 10x Genomics, Pleasanton, CA, USA). The raw sequencing data were demultiplexed to assign reads to individual cells based on their unique cell barcodes and unique molecular identifiers (UMIs). The reads were then aligned using STAR aligner (v2.7.3) [31] to the reference genome (mRatBN7-1.0.0), and a gene expression matrix was generated, quantifying the number of transcripts detected for each gene in each cell. Quality control measures were implemented using the DropletUtils package (v1.16.0) to assess the quality of the cells [32,33]. Cells with greater than 10% mitochondrial content were excluded from further analysis. To identify and remove doublets, Doubletfinder (v2.0.3) was employed [34]. Cells predicted to be doublets were filtered out to enhance the quality of the dataset. To refine the gene expression data further, Soupx (v1.6.2) was used to perform noise correction [35]. The filtered gene expression matrix was imported into R using the Seurat package (v5) [36]. Thereafter, data were downsampled and normalized, and dimensionality reduction was performed using Principal Component Analysis (PCA), and the first 30 principal components were selected for downstream analysis. Clustering was conducted using the Louvain algorithm with a resolution parameter set to 0.5, enabling the identification of distinct cell populations. Cell types were annotated using the ‘FindAllMarkers’ function to identify genes significantly expressed in each cluster, applying a significance threshold of adjusted *p*-value < 0.05. Visualizations were generated using Seurat functions Uniform Manifold Approximation and Projection (UMAP) and dot plots, to depict the clustering of cells and highlight the expression of key marker genes.

### 2.9. Cell–Cell Communication Analysis

Cell–cell communication analysis was performed using the CellChat R package (v2.1.2) to infer interactions from the preprocessed scRNA-seq expression matrix [37]. A CellChat object was created by inputting the expression data and corresponding cell type metadata. Ligand–receptor interactions were identified based on established signaling pathways, allowing for the assessment of overexpressed genes and interactions. Communication probabilities among different cell types were computed to evaluate the strength and directionality of signaling. Finally, the communication networks were visualized using CellChat’s plotting functions to illustrate the interactions and highlight key signaling pathways involved in the cellular communication landscape.

## 3. Results

### 3.1. Characterization of Cellular Clusters and Communication in LV-BMP-2

**Figure 1:**
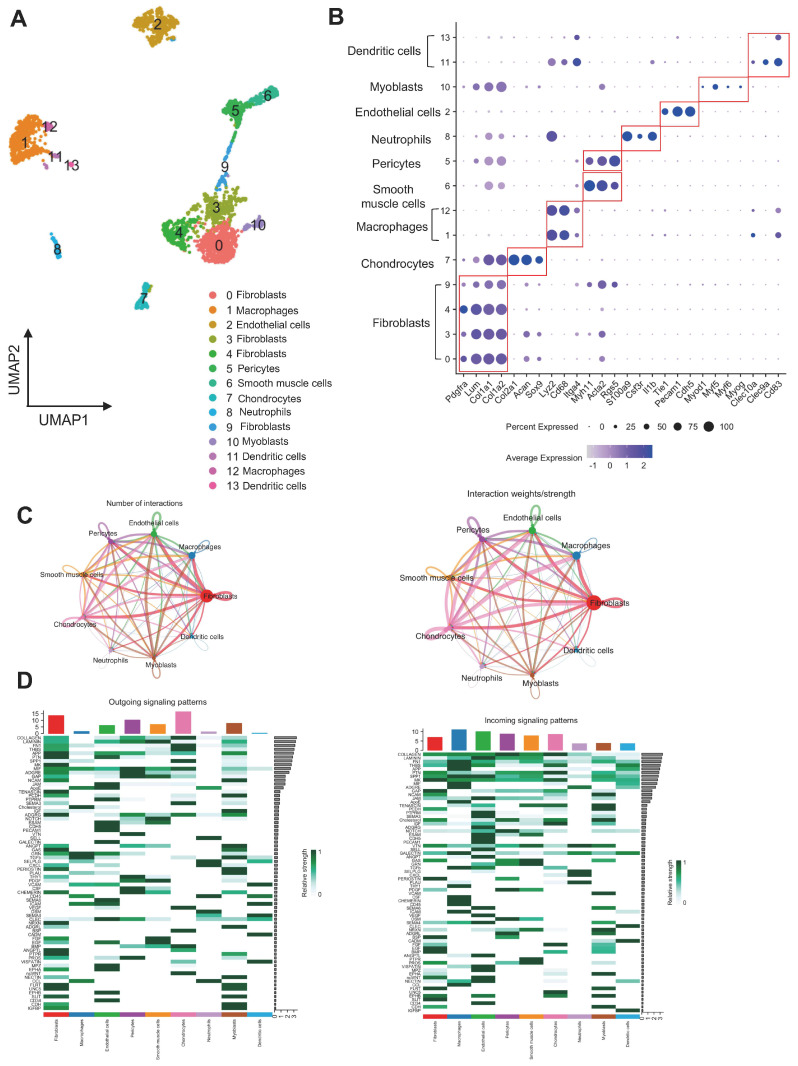
Characterization of cellular heterogeneity in LV-BMP-2. (**A**) UMAP showing the cell populations identified. (**B**) Dot plot showing the expression of marker genes in each cluster. Dot sizes are proportional to the percentage of cells in each cluster expressing the indicated gene. (**C**) Cell communications by ligand–receptor pair analysis. (**D**) Heatmap showing the incoming and outgoing signaling patterns within the different clusters.

In the LV-BMP-2 group, single-cell sequencing yielded 3124 cells for further analysis. To investigate the cellular heterogeneity within these cells, we performed a dimensionality reduction analysis. The uniform manifold approximation and projection (UMAP) plot (Figure 1A) illustrates the distinct clusters identified within the sample, highlighting diverse cell populations. We observed nine distinct clusters, which were annotated as fibroblasts, chondrocytes, macrophages, smooth muscle cells, pericytes, neutrophils, endothelial cells, myoblasts, and dendritic cells. Each cluster exhibits a unique spatial organization, indicating varying cellular characteristics and functions. The distinct clusters were assigned to specific cell types based on the expression of marker genes as shown in Figure 1B.

To gain deeper insight into the intercellular communication dynamics in LV-BMP-2, we employed CellChat analysis, illustrating the incoming and outgoing signaling patterns among different cell types (Figure 1C,D). Notably, we observed that fibroblasts and chondrocytes exhibited strong outgoing signaling suggesting a prominent role in mediating interactions within the cellular microenvironment. In contrast, macrophages demonstrated limited outgoing signals, indicating a more passive role in cell–cell communication. However, macrophages showed predominant incoming signaling patterns. The heatmap reveals that, among several pathways, collagen and BMP pathways were highly activated in chondrocytes, as evidenced by its robust signaling patterns, while BMP pathway showed a higher number of incoming signals for fibroblasts. This differential signaling pattern highlights the functional specificity of cell populations in LV-BMP-2. Together, these results provide clues into the cellular interactions and signaling mechanisms at play within LV-BMP-2, offering a comprehensive overview of the cellular landscape.

### 3.2. Dissecting Cellular Architecture and Communication in rhBMP-2

**Figure 2:**
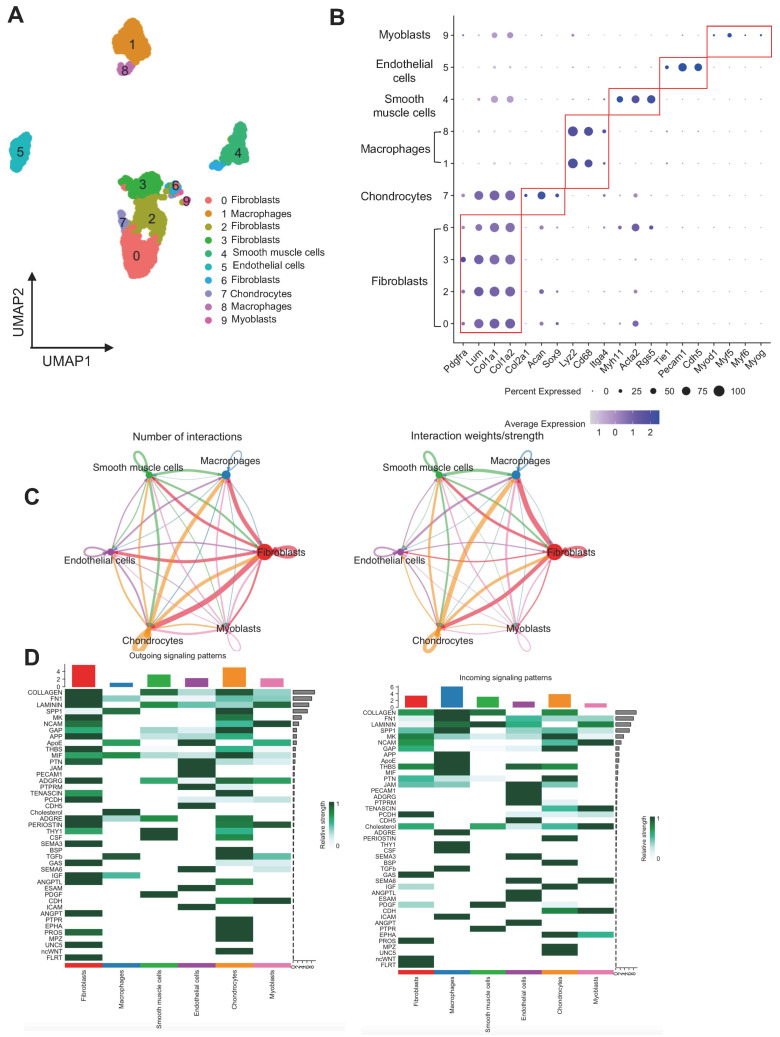
rhBMP-2 shows distinct cellular architecture and cell–cell communication. (**A**) UMAP showing the cell populations identified. (**B**) Dot plot showing the expression of marker genes in each cluster. Dot sizes are proportional to the percentage of cells in each cluster expressing the indicated gene. (**C**) Cell communications by ligand–receptor pair analysis. (**D**) Heatmap showing the incoming and outgoing signaling patterns within the different clusters.

In the rhBMP-2 group, single-cell sequencing yielded 2393 cells for further analysis. The analysis of cellular diversity is illustrated in Figure 2. The UMAP plot reveals several distinct cell clusters (Figure 2A), emphasizing the complexity of the cellular landscape in this sample. In total, we identified six unique clusters and annotated them as fibroblasts, chondrocytes, macrophages, smooth muscle cells, endothelial cells, and myoblasts based on their marker gene expression (Figure 2B). Each cluster displayed a unique arrangement, suggesting distinct functional states and biological roles. To elucidate the intercellular signaling networks present in rhBMP-2, we determined the incoming and outgoing signaling interactions among various cell populations (Figure 2C,D). A prominent feature observed in this analysis was the robust outgoing signals from fibroblasts, indicating a potentially influential role in shaping the cellular environment. Conversely, macrophages displayed higher incoming interactions, suggesting a more receptive function in the signaling landscape. Collagen, laminin, and fibronectin were some of the predominant outgoing pathways from fibroblasts. Macrophages showed predominant incoming signaling for Apolipoprotein E (APOE), transforming growth factor beta (TGFB), and intercellular adhesion molecule (ICAM) pathways.

### 3.3. Bone Regenerative Pathway and Inflammatory Microenvironment Are Altered in LV-BMP2 and rhBMP-2

**Figure 3:**
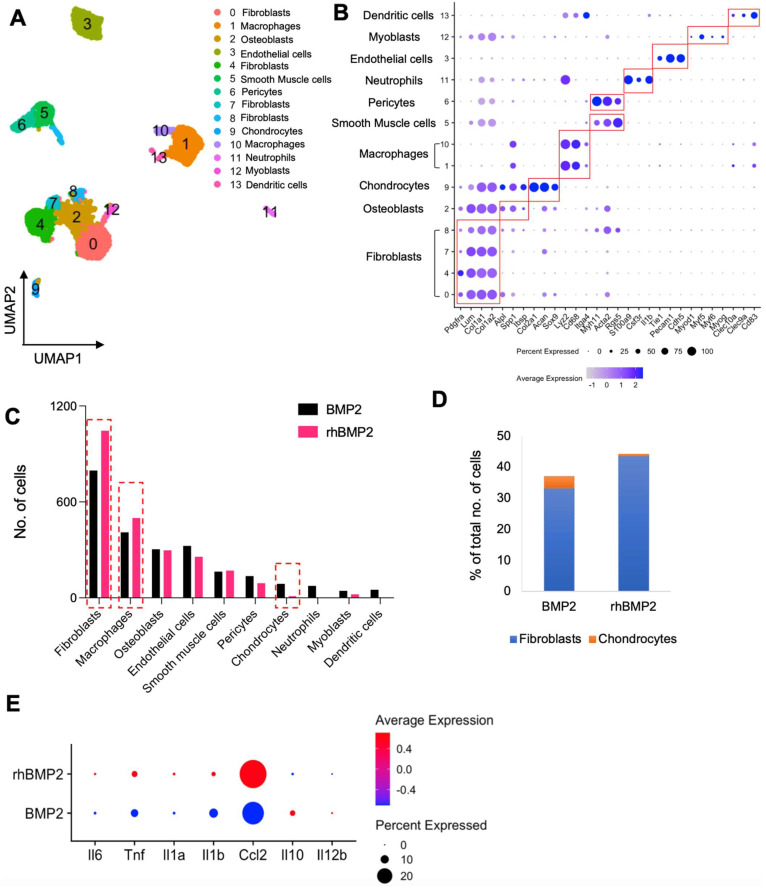
Comparative analysis shows altered bone regenerative pathway and inflammatory microenvironment in LV-BMP2 and rhBMP-2. (**A**) UMAP showing the cell populations identified from the combinatorial analysis of 4786 cells. (**B**) Dot plot showing the expression of marker genes in each cluster. Dot sizes are proportional to the percentage of cells in each cluster expressing the indicated gene. (**C**) The contribution of each sample to each cell type. Red dotted lines indicate the cell populations of interest for further investigation. (**D**) Bar plot showing the ratio of fibroblasts and chondrocytes among all cells in the two samples. (**E**) Dot plot showing the expression of inflammatory and anti-inflammatory genes in macrophage clusters.

The transcriptomic differences between LV-BMP-2 and rhBMP-2 were compared by downsampling and combining the datasets containing 4786 cells (2393 cells from each sample). Computational analysis of this merged dataset revealed 10 unique clusters with distinct gene expression patterns (Figure 3A). These clusters included fibroblasts, osteoblasts, chondrocytes, macrophages, smooth muscle cells, pericytes, neutrophils, endothelial cells, myoblasts, and dendritic cells (Figure 3B). The presence of osteoblasts indicated that the regional gene therapy LV-BMP-2 induces osteoinductive changes. Cell distribution analysis showed that LV-BMP-2 samples had a higher number of chondrocytes as compared to rhBMP-2 (Figure 3C). In addition, rhBMP-2 had more macrophages as compared to LV-BMP-2. To assess the quality of bone formation, the ratio of chondrocytes to fibroblasts were investigated (Figure 3D). We observed that rhBMP-2 had a higher fibroblast to chondrocyte ratio as compared to LV-BMP-2. Previous studies have reported that nonunions contain a significant proportion of fibroblasts which fail to ossify [38,39]. To gain further insight into the immunomodulation and inflammatory activity at the bone repair site, we checked the expression of pro-inflammatory and anti-inflammatory cytokines in the macrophage clusters specifically (cluster 1 and 10) (Figure 3A,B). Interestingly, well known pro-inflammatory cytokines like interleukin 6 (IL6), tumor necrosis factor alpha (TNFA), IL1A, IL1B, and C-C Motif Chemokine Ligand 2 (CCL2) were highly expressed in rhBMP-2 as compared to LV-BMP-2 (Figure 3E). However, anti-inflammatory cytokines like IL10 and IL12B were observed to be expressed more in LV-BMP-2 suggesting that this gene therapy is potentially safe from a clinical standpoint (Figure 3E). Overall, these transcriptomic dissimilarities suggest differences in the bone regenerative pathways as well as inflammatory microenvironment between LV-BMP-2 and rhBMP-2 treatment.

### 3.4. Comparative Analysis of Intercellular Communication in LV-BMP-2 and rhBMP-2

**Figure 4:**
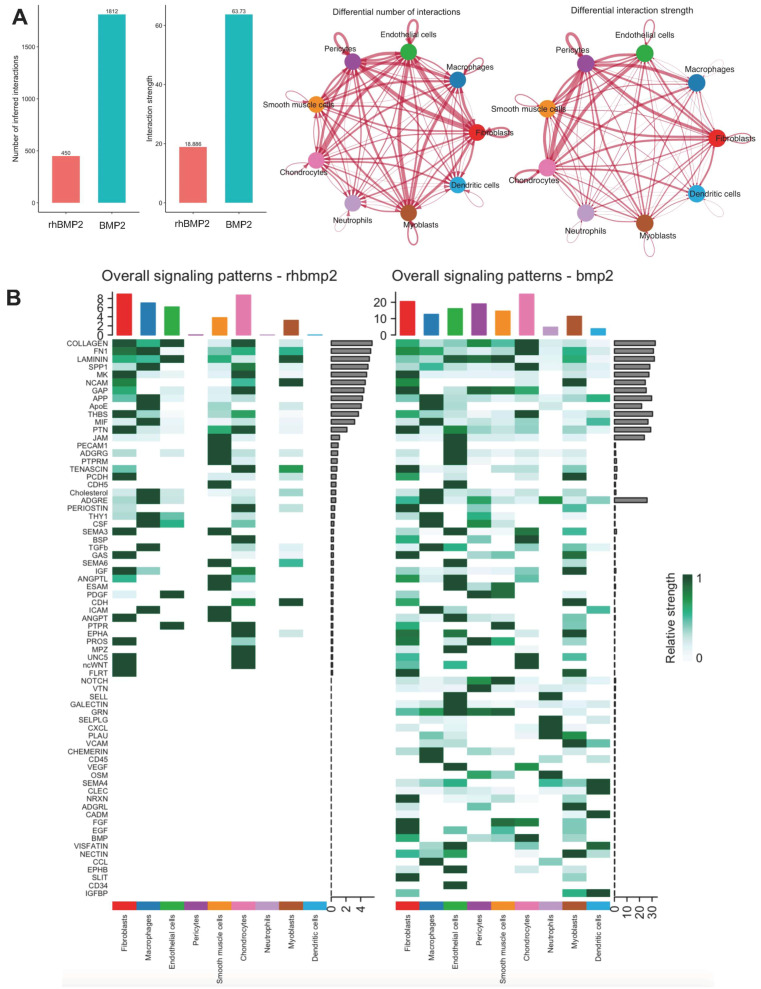
Comparative intercellular communication in LV-BMP2 and rhBMP-2. (**A**) Plots showing the total number of interactions and interaction strength of the inferred cell–cell communication networks. (**B**) Heatmap comparing the overall signaling pattern between the two samples allowing to identify signaling pathways/ligand–receptors that exhibit different signaling patterns.

To evaluate the differences in intercellular communication between gene therapy and rhBMP-2, we performed a comparative analysis of CellChat outputs. The results revealed distinct variations in the signaling networks across the two samples, indicating differential roles of cell types in communication. The gene therapy group showed a significantly higher number of interactions with a higher interaction strength (Figure 4A) compared to rhBMP-2 which suggests that there is increased cellular crosstalk in gene therapy, leading to more extensive intercellular communication. Several pathways including neurogenic locus notch homolog protein (NOTCH), fibroblast growth factor (FGF), BMP, and epidermal growth factor (EGF) were distinctly enriched in LV-BMP-2 compared to rhBMP-2 (Figure 4B). These differences may reflect underlying biological processes playing an active role in modulating the tissue microenvironment. Overall, this comparative analysis of CellChat outputs between the two strategies underscores two distinct cellular communication landscapes.

### 3.5. Reclustering Fibroblasts and Macrophage Clusters Reveals Distinct Cellular Subpopulations and Marker Gene Signatures

**Figure 5:**
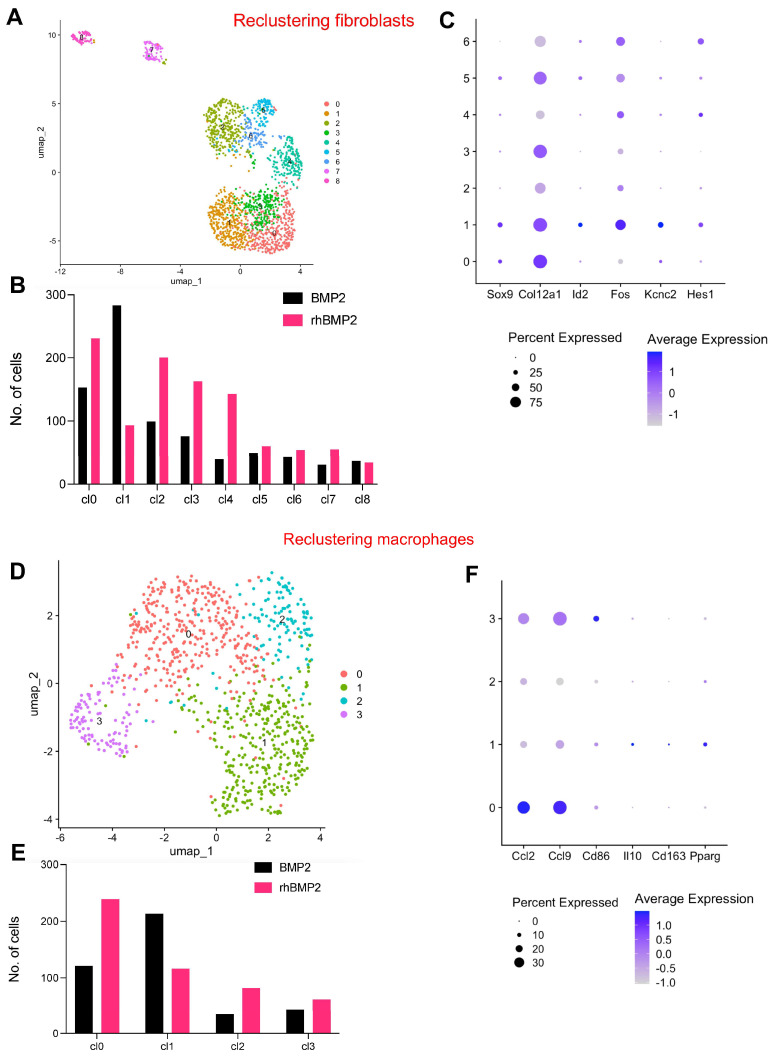
Reclustering and characterizing fibroblasts and macrophages. (**A**) UMAP showing the 9 distinct reclustered fibroblast populations. (**B**) Bar plot showing the distribution of the populations. (**C**) Dot plot showing the expression of marker genes in the clusters. Dot sizes are proportional to the percentage of cells in each cluster expressing the indicated gene. (**D**) UMAP showing the 3 distinct reclustered macrophage populations. (**E**) Bar plot showing the distribution of the populations. (**F**) Dot plot showing the expression of marker genes in the clusters. Dot sizes are proportional to the percentage of cells in each cluster expressing the indicated gene.

To achieve a higher resolution of the cellular diversity within our downsampled, combined dataset, we performed reclustering for the fibroblast and macrophage clusters. Reclustering for chondrocytes did not identify definitive clusters due to the overall low numbers of chondrocytes. This analysis allowed us to capture finer subpopulations that were not fully distinguishable in the initial analysis. We identified nine distinct subclusters for fibroblasts (Figure 5A,B), whereas, for macrophages, we obtained four distinct clusters (Figure 5D,E), highlighting previously unrecognized heterogeneity within the sample. Following reclustering, we conducted a comprehensive analysis of marker genes specific to each subpopulation (Figure 5C,F). For fibroblasts, cluster 1 showed that the enrichment in LV-BMP-2 and the marker genes corresponding to this cluster was associated with neurogenesis (expressing *Id2*, *Fos*, *Kcnc2*, and *Hes1*). Similarly, for macrophages, cluster 0 was predominant in rhBMP-2 and showed gene signatures corresponding to M1 phenotype (expressing *Ccl2*, *Ccl9*, and *Cd86*), whereas cluster1 showed a higher expression for M2 phenotype (expressing *Il10*, *Cd163*, and *Pparg*). This refined clustering and marker gene analysis provided a more granular view of the cellular landscape, allowing us to identify specialized cell types or functional states that were masked in the initial analysis. The identification of these marker genes lays the foundation for the further functional characterization of these subpopulations, including potential roles in mediating fibrosis and the immune response.

## 4. Discussion

*Ex vivo* regional gene therapy is a promising bone-tissue-engineering strategy as it can provide three essential components: a population of osteogenic cells, a sustained osteoinductive stimulus, and an osteoconductive scaffold. Our research group has focused on an approach that utilizes lentiviral transduced adipose-derived MSCs that overexpress BMP-2 (LV-BMP-2). The development of this strategy was initially motivated by a desire to overcome limitations associated with the delivery, duration of activity, and side effects of rhBMP-2. The use of MSCs transduced with a lentiviral vector allows for sustained BMP-2 expression *in vivo*. In addition, the presence of MSCs and their interactions with the secreted BMP-2 may contribute to bone healing either directly through MSC differentiation, or indirectly through alterations in the microenvironment. To the best of our knowledge, this study provides the first detailed characterization of the cell populations and molecular mechanisms associated with *in vivo* bone regeneration in two seemingly similar BMP-2 mediated treatment approaches: LV-BMP-2 gene therapy with adipose-derived MSCs and rhBMP-2. By utilizing a single-cell RNA-sequencing approach to evaluate these dynamic biologic environments, we were able to identify treatment-specific differences in cell populations and transcriptomics. These findings support that LV-BMP-2 gene therapy may improve bone healing through unique pathways related to enhanced chondrogenesis, reduced fibrosis, and favorable immunomodulation with an increased presence of anti-inflammatory macrophages and cytokines when compared to rhBMP-2.

The biology of bone healing is a highly complex and regulated process involving multiple cell types and the expression of numerous genes [40,41,42]. In concordance with the literature, we found that hematoma tissue in the setting of a segmental femoral bone defect is a heterogenous tissue with numerous cell types engaging in complex crosstalks. In both treatment groups, we observed the presence of expected cell types including osteoblasts, chondrocytes, fibroblasts, smooth muscle cells, and pericytes, as well as multiple immune cell types. Cell-chat analysis, which reveals intricate cell–cell communication networks and identifies key signaling pathways that contribute to the functional dynamics of the cell populations within samples, showed highly active and inter-related cell signaling networks during bone healing.

Endochondral ossification is a type of bone healing that occurs via a cartilage intermediate. It is the predominant process of healing in segmental long bone defects and was expected in both treatment groups [40,43]. A cell distribution analysis revealed that the LV-BMP-2 treatment group had a higher number of chondrocytes compared to the rhBMP-2 group, which suggests a more robust endochondral ossification response [44,45]. While we were unable to perform lineage tracing, there are a few plausible explanations for this finding. Adipose-derived MSCs implanted as part of the LV-BM-2 approach retain multipotency and may differentiate into chondrocytes under the local effects of BMP-2 or other cytokines, particularly in the initially hypoxic setting of a segmental bone defect [46]. This finding is supported by upregulated BMP pathways in the chondrocytes of our LV-BMP-2 samples. Alternatively, MSC mobilization (i.e., homing) to the site of injury may be increased in the LV-BMP-2 group, providing an additional source of osteoprogenitors [47]. A marker gene analysis revealed increased the expression of genes previously shown to act as MSC homing molecules including *bmps*, *tgfb1*, and *vegf* [48,49]. In addition to the increased number of chondrocytes, this cell population also showed increased the signaling of multiple factors associated with cartilage growth and maturation including BMPs, VEGF, FGF, TGFB1, FN1, and SEMA3 [4,50,51,52,53]. Together, these results support that LV-BMP-2 gene therapy mediates improved endochondral ossification in the early stages of healing compared to rhBMP-2, and may help to explain why improved bone quality has been seen in prior comparative preclinical studies [16,54]. Further study is needed to more clearly define the role of implanted and host MSCs in newly forming cartilage.

Fibrosis is one of the major pathological features of non-union, a condition which occurs when a bone fails to heal appropriately [55,56]. Fibrosis is mediated by fibroblasts, but these cells also have a diverse role in various cellular processes. In our study, fibroblasts were the most abundant cell type present in both groups but were more numerous in the rhBMP-2 group, accounting for approximately 45% of all cell types. Our marker gene analysis demonstrated that fibroblasts in the rhBMP-2 group had an upregulated expression of collagen and extracellular matrix protein pathways, reflecting a more pro-fibrotic state. Fibroblasts could be further divided into eight clusters; the marker gene analysis showed transcriptomic heterogeneity within the clusters that was associated with specific tissue types including skeletal muscle, vascular smooth muscle, tendons/ligaments, and neurons. These data underscore the multiple roles of fibroblasts in injury response and repair. In one cluster, the LV-BMP-2 group had a three-fold increase in the number of cells compared to rhBMP-2; this cluster showed an enrichment of pathways related to neurogenesis. Nerve function has been previously shown to have an important role in fracture healing. Animal studies of limb denervation demonstrate abundant but disorganized fracture callus, whereas the early reinnervation of calluses is associated with a greater endochondral response [57,58,59]. Moreover, BMP-2 has been shown to possess neurotrophic functions in the peripheral nervous system, and its expression may improve healing after nerve injury [60,61]. While these are preliminary findings, the results suggest a potential novel pathway through which the sustained BMP-2 expression in LV-BMP-2 gene therapy improves endochondral bone healing and reduces the risk of nonunion via enhanced neurogenesis.

Inflammatory infiltration in the early stages of bone injury is characterized by the production of various pro-inflammatory cytokines and the recruitment of macrophages and other mediators of the innate immune system [41]. In our study, the rhBMP-2 group demonstrated an increased number of macrophages in the fracture hematoma. These macrophages also differentially expressed pro-inflammatory cytokines including IL1B, IL6, and TNFA, suggesting phenotypic polarization towards an M1 phenotype [62,63]. Excessive inflammation in the setting of bone healing is mediated, in part, by the factors described above and results in the inhibition of chondrogenesis and osteogenesis [43,64,65,66]. In contrast, macrophages in the LV-BMP-2 group expressed anti-inflammatory genes including IL10 and IL12, and macrophage clustering revealed an increased prevalence of gene pathways consistent with a M2 phenotype. Differences in the inflammatory microenvironment between the two treatment groups may be explained by the immunomodulatory effects of MSCs and BMP-2 on macrophages. MSCs have wide-ranging and well-characterized effects on osteoimmunology. In macrophages, MSCs induce a transition towards a M2 phenotype [67]. BMP-2 has also been shown to have an immunoregulatory role, activating macrophages but inhibiting the expression of M1 marker genes, which in turn improves the osteogenic differentiation of MSCs [62,68]. Collectively, these results suggest a beneficial immunomodulatory effect of LV-BMP-2 for bone healing. While further study of these pathways is needed, it is a particularly promising feature of gene therapy as a massive inflammatory response is thought to be responsible for many of the clinical side effects associated with rhBMP-2 [8,69].

In light of the above findings, it is noteworthy that our analysis also revealed a small population of neutrophils that was exclusively present in the LV-BMP-2 group. Inflammatory infiltration in the early stages of fracture or bone defect healing includes neutrophils and would be expected in both treatment groups [41]. However, their presence peaks within days of initial injury and declines rapidly thereafter [70]. The persistence of neutrophils in the LV-BMP-2 fracture hematoma at 2 weeks may be explained by the immunomodulatory effect of MSCs, as prior work has shown that MSCs prolong the neutrophil lifespan [71]. Additionally, we cannot discount the possible contribution of an increased innate immune response related to the immunogenicity of viral proteins co-expressed in the transduced cells. Neutrophils may be recruited to the site of gene delivery to manage inflammation or infection risks, especially with viral vectors that might be perceived as pathogenic by the immune system. Gene therapy has a higher potential to activate immune cells, including neutrophils, due to the involvement of viral vectors and genetic material, while recombinant therapy may bypass this innate immune response unless other factors (e.g., protein impurities or immune hypersensitivity) are involved [12,72].

The findings of the present study should be interpreted in the context of its limitations. This study as well as our prior work utilized a rat critical-sized femoral defect model, which is the most commonly used preclinical model for bone healing. Twelve-week-old rats were used, which are skeletally mature young adults. Both bone healing and the response to BMP-2 are affected by age, and, thus, our results may not be generalizable to older rats receiving these therapies [73,74,75]. Further study is required to address whether age influences these data. In addition, BMP-2 has species-specific concentrations to induce osteogenesis, which is lower in rats than in humans [76]. Thus, we may have been unable to detect dose-dependent pathways that have been implicated in the side effects of rhBMP-2 used in humans [8]. However, the aim of the present study was to study the cell populations and pathways associated with two different BMP-2 mediated therapeutic approaches; our cell “dose” for the gene therapy group (i.e., 5 million transduced MSCs) and the dose of rhBMP-2 were based on prior work showing these doses were sufficient for segmental defect healing in our model. We have tried to understand the cellular heterogeneity and the potential effects at a transcriptomic level. However, it would be important to further validate the observations at a protein level using various functional assays (e.g., Western blot, and enzyme-linked immunosorbent assay) in order completely understand the complex mechanisms responsible for bone defect healing.

Despite these limitations, the current study is valuable as a transcriptomic analysis at the single-cell resolution of regional gene therapy and rhBMP-2 strategies enabled us to gain unprecedented insights into the cellular heterogeneity and specific gene expression profiles within them, ultimately enhancing our understanding of the unique biological processes promoted by each therapy. Additional strengths of the study include rigorous bioinformatic analysis and quality control measures to ensure appropriate between-treatment comparisons. Future studies using higher doses of rhBMP-2 and evaluating multiple time-points after bone defect treatment will provide a more complete understanding of the dynamic changes in cells and gene expression that occur in response to injury and BMP-2 mediated therapies.

## 5. Conclusions

In summary, the application of single-cell RNA-sequencing technology to BMP-2-mediated bone regeneration in a rat critical-sized femoral defect yielded a detailed picture of cellular and transcriptomic heterogeneity and led to the identification of treatment-specific differences between (1) *ex vivo* regional gene therapy with lentiviral transduced adipose-derived mesenchymal stem cells with a BMP-2 transgene and (2) recombinant human BMP-2. Our results suggest that gene therapy result may promote unique chondrogenic, anti-fibrotic, and anti-inflammatory pathways that results in improved bone regeneration compared to a clinically approved treatment, rhBMP-2.

## Data Availability

The data that support the findings of this study are available from the corresponding author upon reasonable request.

## Supplementary Materials

The following supporting information can be downloaded at: https://www.mdpi.com/article/10.3390/bioengineering12010029/s1, Figure S1: BD FACSDiva 8.5.
