# Supplementary figures and images for: Ex Vivo Regional Gene Therapy Compared to Recombinant BMP-2 for the Treatment of Critical-Size Bone Defects: An In Vivo Single-Cell RNA-Sequencing Study
Source: Bioengineering (Basel). 2025 Jan 1;12(1):29. doi: 10.3390/bioengineering12010029 (PMC11762083; doi:10.3390/bioengineering12010029)

# BD FACSDiva 8.5

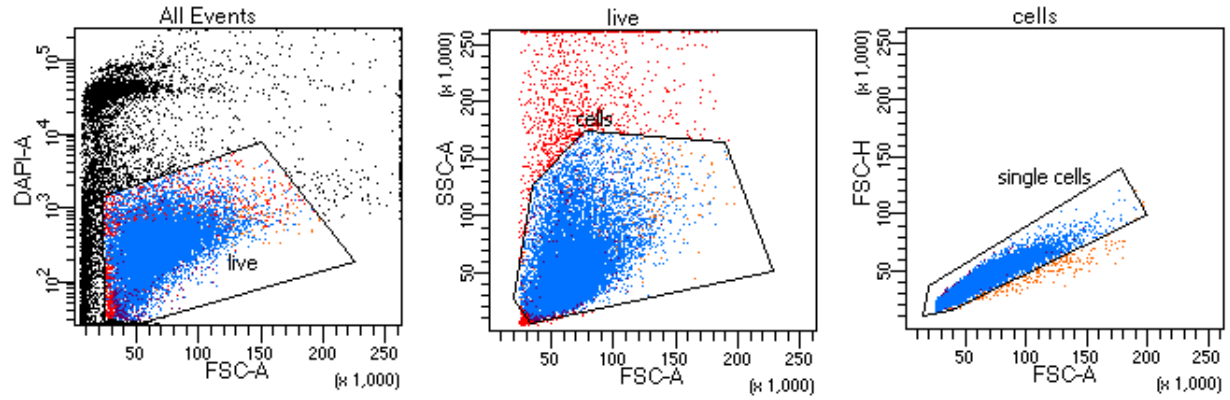

Tube: Tube\_005

| Population   | #Events | %Parent | %Total |
|--------------|---------|---------|--------|
| All Events   | 25,000  | ####    | 100.0  |
| live         | 15,460  | 61.8    | 61.8   |
| cells        | 13,931  | 90.1    | 55.7   |
| single cells | 13,682  | 98.2    | 54.7   |
| P1           | 11,492  | 84.0    | 46.0   |

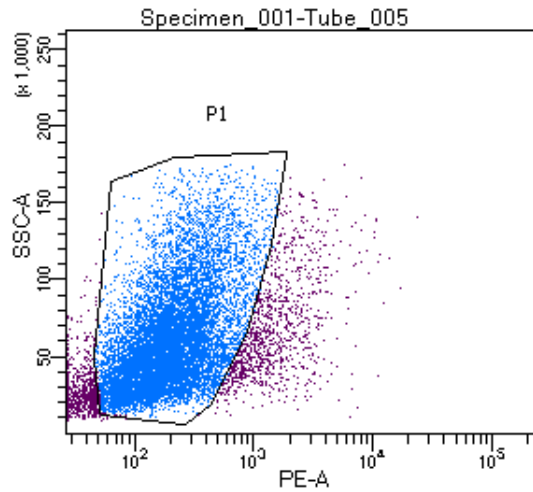

Supplement: Supplementary file 1 [file bioengineering-12-00029-s001.zip › bioengineering-3334663-supplementary.pdf]
